# Comparative Study of Postoperative Healing Outcomes With and Without Medicated Cavity Packing Following Canal Wall Down (CWD) Mastoidectomy

**DOI:** 10.7759/cureus.81837

**Published:** 2025-04-07

**Authors:** Mani Mala, Richi Sinha, Rakesh K Singh

**Affiliations:** 1 Otolaryngology - Head and Neck Surgery, Indira Gandhi Institute of Medical Sciences, Patna, IND

**Keywords:** canal wall down mastoidectomy, chronic suppurative otitis media, epithelialization, ointment pack, postoperative healing

## Abstract

Objective

The objective of this study was to evaluate the effect of postoperative medicated mastoid cavity packing on healing outcomes following canal wall down (CWD) mastoidectomy compared to no packing.

Methods

This prospective observational study included 125 patients undergoing CWD mastoidectomy, matched for age and sex, and assigned to two groups. Group A (n=62) received medicated mastoid cavity packing containing ofloxacin, ornidazole, clobetasone propionate, and itraconazole on postoperative days 0, 10, and 20. Group B (n=63) received no postoperative packing. Healing outcomes were evaluated and compared between the groups on postoperative days 45, 75, and 105. A p-value of <0.05 was considered statistically significant.

Results

The groups were comparable in age and sex distribution. The packed group demonstrated significantly higher rates of complete epithelialization on day 45 (48.4% vs. 15.9%, p < 0.0001) and day 75 (72.6% vs. 49.2%, p = 0.007), with no difference observed by day 105. Graft uptake duration was similar between groups (76.94 days in Group A and 76.19 days in Group B). Granulation tissue, mucosal folds, and otorrhea were more frequent in the non-packed group (25.4%, 6.3%, and 4.8% vs 12.9%, 3.2%, and 3.2%, respectively) during early follow-up, though not statistically significant. Mean epithelialization time was significantly shorter in the packed group (84.0 vs. 92.8 days, p = 0.001).

Conclusion

Medicated mastoid cavity packing promotes earlier epithelialization following CWD mastoidectomy and is associated with a lower incidence of granulation tissue without affecting graft uptake. It offers particular benefits in low-resource settings by enhancing healing and reducing follow-up needs. Larger studies are warranted to establish standardized postoperative care protocols.

## Introduction

Chronic otitis media (COM) remains a significant public health issue, particularly in developing countries, with an estimated prevalence ranging from 1% to 46%, depending on socio-demographic factors [1,2]. In India, the prevalence is approximately 4%, as reported by the WHO/CIBA Foundation workshop in 1996, highlighting its substantial disease burden [3,4]. COM is characterized by a chronic infection of the middle ear cleft, including the eustachian tube, middle ear, and mastoid cavity, with a non-intact tympanic membrane and persistent otorrhea for at least two weeks. If inadequately treated, COM can result in serious complications such as hearing loss, labyrinthitis, facial nerve paralysis, and intracranial infections [5].

Canal wall down (CWD) mastoidectomy is a well-established surgical approach for the management of the squamosal type of COM. The technique facilitates complete disease clearance and reduces the risk of recurrence by creating a self-cleaning mastoid cavity. However, postoperative healing is often protracted due to delayed epithelialization, persistent otorrhea, granulation tissue formation, and meatal stenosis [6]. Postoperative ear packing has long been a standard practice in otologic surgery to promote healing, prevent infection, and minimize complications. While several studies have evaluated different packing materials and medications, there is still no consensus regarding the necessity of packing, the optimal material or drug combination, the mode of application, or the ideal duration of use [7,8].

A recent animal study by Liu et al. (2023) demonstrated that gauze packing containing a bacteriostatic agent reduced the postoperative need for topical and systemic antibiotics following otitis media surgery in a rat model [9]. In contrast, Javed et al. (2015), in a pilot study involving 32 human subjects, found no significant difference in aural symptoms or healing outcomes between patients with and without postoperative ear packing [10]. While several studies have examined the role of packing in the external auditory canal and middle ear [11,12], there remains a paucity of evidence specifically addressing the impact of mastoid cavity packing during the postoperative follow-up phase of CWD mastoidectomy.

In developing countries, where poor hygiene and antibiotic resistance contribute to delayed healing, the use of medicated mastoid packing could offer a cost-effective strategy to accelerate recovery and reduce complications. Given the lack of standardized guidelines on postoperative mastoid packing, this study aims to evaluate the effectiveness of medicated mastoid cavity packing compared to no packing in postoperative cases of CWD mastoidectomy. By analyzing key healing parameters, such as epithelialization, granulation tissue formation, graft uptake, and meatal narrowing, we seek to develop evidence-based recommendations to improve postoperative outcomes and cost-efficiency in CWD mastoidectomy care.

## Materials and methods

Study design and setting

This prospective observational study was conducted in the Department of Otorhinolaryngology at Indira Gandhi Institute of Medical Sciences, Patna, India, between March 2024 and December 2024. The study involved human subjects and was approved by the Institutional Ethics Committee (Approval No. 1470/IEC/IGIMS/2024, dated 20/03/2024). Written informed consent was obtained from all participants. The study was conducted in accordance with the ethical principles of the World Medical Association Declaration of Helsinki.

Study participants

Patients aged five years and above undergoing CWD mastoidectomy who provided written informed consent were included. Exclusion criteria were uncontrolled hypertension, diabetes mellitus, immunodeficiency, anemia, acute illness, history of substance use, prior ear surgery, or inability to comply with postoperative follow-up.

Sample size and sampling

The sample size was calculated based on a 2015 pilot randomized controlled trial by Javed et al. [10], which compared the outcomes of external ear packing versus no packing following middle ear surgery. To achieve 90% statistical power, a total of 125 patients undergoing CWD mastoidectomy were included in the study. Two age- and sex-matched cohorts were prospectively formed. Group A (n = 62) received medicated mastoid cavity packing using ointment-soaked ribbon gauze placed over gelfoam, while Group B (n = 63) received only gelfoam without additional packing. Patients were assigned alternately to the two groups based on age and sex matching to minimize potential confounding variables and ensure that these baseline characteristics did not influence postoperative healing outcomes.

Clinical protocol and intervention

A detailed medical history and comprehensive clinical examination, including otoendoscopy, were performed for all 125 patients, and findings were systematically documented. Once CWD mastoidectomy was planned, preoperative investigations, including routine blood tests, chest X-ray, electrocardiogram, and radiological imaging (as indicated) were carried out. A pre-anesthetic evaluation was completed for all patients, and those meeting the surgical criteria underwent CWD mastoidectomy performed by a single experienced ENT surgeon to ensure procedural consistency.

**Figure 1:**
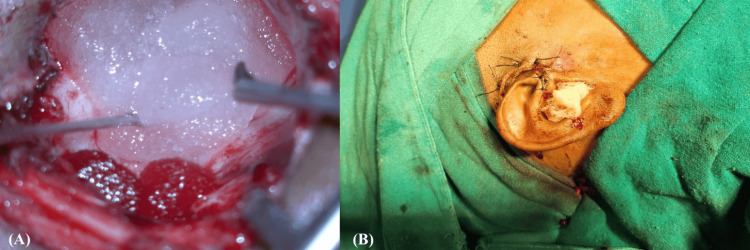
Intraoperative photographs (A) The conventional packing of the mastoid cavity with gel foam; (B) Packing of the mastoid cavity with ribbon gauze impregnated with combination ointment

Following surgery, antibiotic-soaked gelfoam was placed in the mastoid cavity and middle ear in all patients. Subsequently, patients were divided into two age- and sex-matched cohorts. In Group A (n = 62), the mastoid cavity was additionally packed with sterile ribbon gauze impregnated with a combination ointment containing ofloxacin (0.75% w/w), ornidazole (2% w/w), clobetasone propionate (0.05% w/w), and itraconazole (1% w/w), applied under aseptic precautions (Figure 1). In Group B (n = 63), no ribbon gauze packing was applied, and only gelfoam was used. Mastoid dressing was applied in all cases, and patients were discharged on postoperative day (POD) 5 with a course of oral antibiotics.

On the first follow-up (POD 10), the initial pack and gelfoam were removed. Group A was re-packed using the same ointment-impregnated gauze, whereas Group B patients were prescribed ear drops containing beclomethasone dipropionate (0.025% w/v), neomycin sulfate (0.5% w/v), and clotrimazole (1.0% w/v), administered as two drops three times daily until the third follow-up. Systemic antibiotics were discontinued in both groups two weeks post-surgery. Finally, the same procedure was repeated on POD 20. All patients were instructed to monitor for signs of infection, including fever, malaise, headache, otorrhea, foul odor, or postauricular swelling, and to report immediately for early pack removal and appropriate intervention if needed. Any such events were documented to evaluate the safety and tolerability of repeated packing. Lastly, the pack was removed on POD 30.

Data collection

Structured follow-up assessments were conducted on POD 45 (15 days after final pack removal), POD 75, and POD 105. Each follow-up included an otoendoscopic examination, and postoperative outcomes were assessed by a blinded evaluator unaware of the patient’s group allocation. Parameters evaluated included the presence of discharge, granulation tissue, mucosal folds, meatal narrowing, graft uptake, and the degree and rate of epithelialization. The collected data were analyzed to determine the impact of medicated mastoid cavity packing on postoperative healing following CWD mastoidectomy.

Statistical analysis

All statistical analyses were conducted using Microsoft Excel (v16.89; Microsoft Corporation, Redmond, WA, US) and XLSTAT (v2024.4.0.1424; Lumivero, LLC, Denver, Colorado, US). Descriptive statistics were used to summarize the surgical outcomes, including graft healing rates and postoperative recovery parameters. The Shapiro-Wilk test was applied to assess the normality of continuous variables. For categorical variables, such as discharge, graft uptake, epithelialization, mucosal fold formation, granulation tissue formation, and meatal narrowing, data were expressed as frequencies and percentages. For continuous variables like age, data were expressed as mean ± standard deviation (SD). Comparisons between groups were made using the chi-square test or Fisher’s exact test, as appropriate, for categorical variables. The Mann-Whitney U test was used to compare ages between groups due to non-normal distribution. Kaplan-Meier survival analysis was employed to compare epithelialization and graft uptake rates between the two groups, with time to complete epithelialization and well-taken graft considered as endpoints. Patients who did not achieve the endpoint within the study period or were lost to follow-up were censored. Mean times to epithelialization and graft uptake, along with 95% confidence intervals (CIs), were calculated for each group, and the log-rank test was used to assess statistical significance. A p-value of < 0.05 was considered statistically significant.

## Results

Demographic characteristics

The demographic characteristics of the study participants are summarized in Table 1. The mean age was comparable between Group A (with pack) and Group B (without pack) (25.76 ± 13.87 vs. 22.98 ± 11.75 years, respectively; p = 0.322). The distribution of males and females was also similar between groups, with 28 (45.2%) males in Group A and 26 (41.3%) in Group B (p = 0.661). Thus, no statistically significant demographic differences were found between the two groups.

**Table 1:** Demographic characteristics of the study participants The p-values were calculated using the Mann-Whitney U-test for age and the chi-square test for sex distribution. A p-value of < 0.05 was considered statistically significant. SD: standard deviation

Characteristic	Group A (with pack), n = 62	Group B (without pack), n = 63	p-value
Age (in years), mean ± SD	25.76 ± 13.87	22.98 ± 11.75	0.322
Male (%)	28 (45.2%)	26 (41.3%)	0.661
Female (%)	34 (54.8%)	37 (58.7%)	0.661

Postoperative local complications

**Figure 2:**
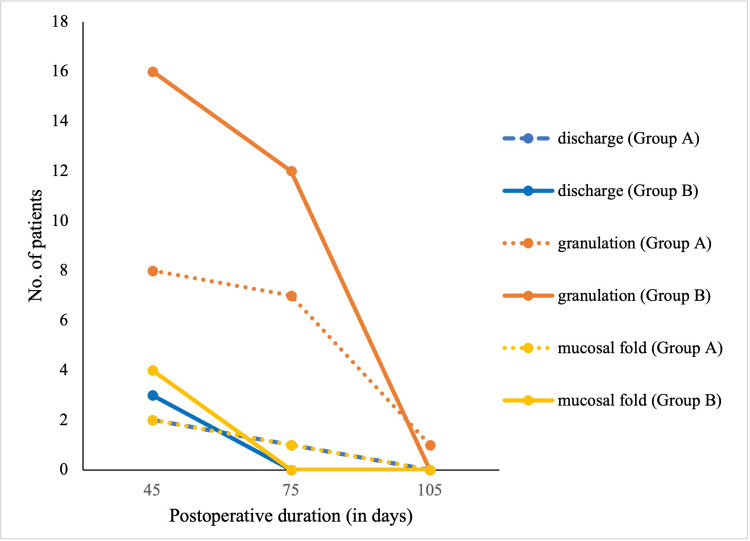
Line diagram showing the postoperative incidence of discharge, granulation tissue formation, and mucosal fold development in Group A (with pack) and Group B (without pack) over time

The incidence of postoperative complications, including ear discharge, granulation tissue, and mucosal fold formation, was evaluated at POD 45, 75, and 105 (Figure 2). At POD 45, granulation tissue was present in 16 patients (25.4%) in Group B, compared to 8 patients (12.9%) in Group A (p = 0.122). Mucosal fold formation and ear discharge showed no statistically significant differences between the groups.

At POD 75, granulation was noted in 12 patients (19.0%) in Group B and 7 patients (11.3%) in Group A (p = 0.338). Mucosal fold and discharge incidences remained low and comparable in both groups. By POD 105, most complications had resolved, with granulation tissue present in only one patient (1.6%) in Group A and none in Group B (p = 0.496). No cases of mucosal fold or discharge were observed at this time point. It is worth mentioning that during our study period, none of the cases suffered from postoperative meatal stenosis in either group. Postoperative healing outcomes across various parameters are summarized in Table 2.

**Table 2:** Postoperative follow-up data at days 45, 75, and 105 comparing Groups A and B at each follow-up point for the incidence of postoperative healing and local complications Data are presented as absolute frequencies (n). p-values were calculated using the chi-square test or Fisher’s exact test, as appropriate. * p < 0.05 was considered statistically significant. Graft uptake was 100% by postoperative day (POD) 75 in both groups; hence, no statistical comparison was made.

Follow-up	Parameter	Group-A (with pack), n = 62	Group-B (without pack), n = 63	p-value
POD 45	Discharge (%)	2 (3.2%)	3 (4.8%)	1.000
	Granulation (%)	8 (12.9%)	16 (25.4%)	0.122
	Mucosal fold (%)	2 (3.2%)	4 (6.3%)	0.679
	Complete epithelization (%)	30 (48.4%)	10 (15.9%)	<0.0001*
	Graft uptake (%)	54 (87.1%)	58 (92.1%)	0.538
POD 75	Discharge (%)	1 (1.6%)	0	0.496
	Granulation (%)	7 (11.3%)	12 (19.0%)	0.338
	Mucosal fold (%)	1 (1.6%)	0	0.496
	Complete epithelization (%)	45 (72.6%)	31 (49.2%)	0.007*
	Graft uptake (%)	62 (100%)	63 (100%)	-
POD 105	Discharge (%)	0	0	-
	Granulation (%)	1 (1.6%)	0	0.496
	Mucosal fold (%)	0	0	-
	Complete epithelization (%)	56 (90.3%)	60 (95.2%)	0.473
	Graft uptake (%)	62 (100%)	63 (100%)	-

Complete epithelialization and graft uptake

**Figure 3:**
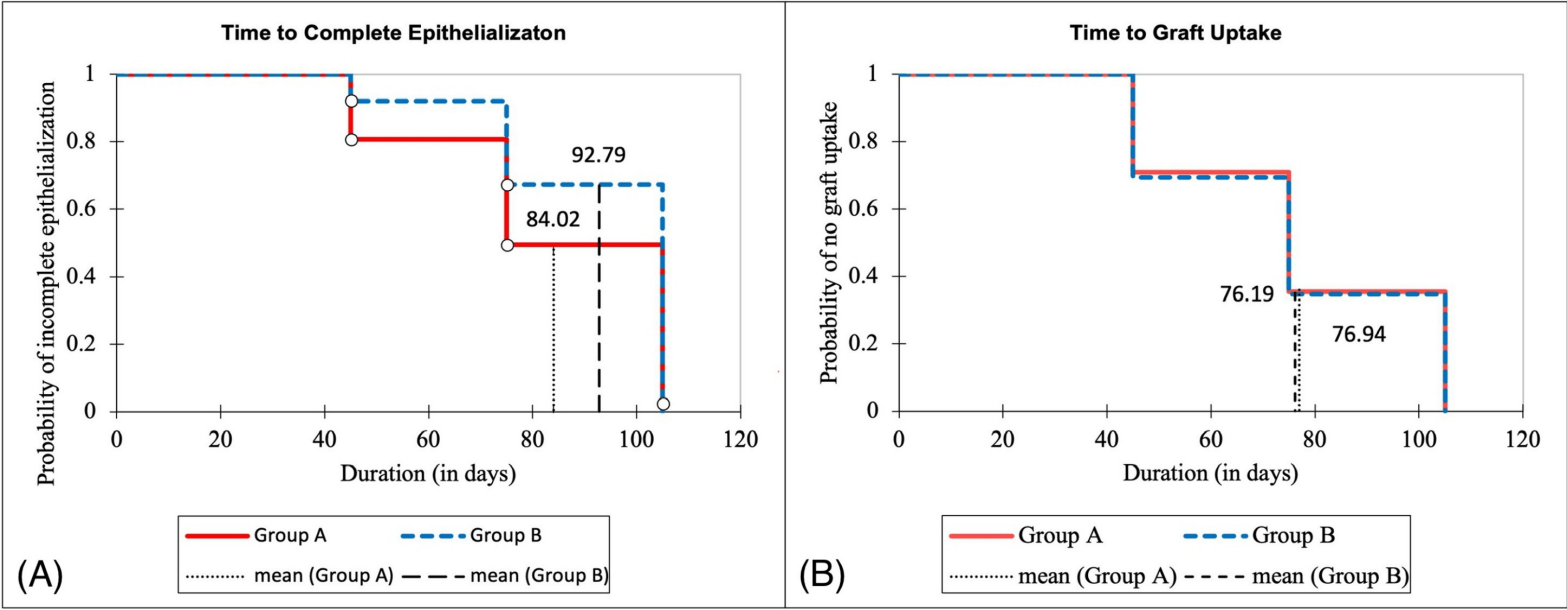
Kaplan–Meier survival curves (A) Time to complete epithelialization in Group A (with pack) and Group B (without pack); (B) Time to graft uptake in Group A (with pack) and Group B (without pack)

Kaplan-Meier survival analysis revealed the time to complete epithelialization and graft uptake in the two groups (Figure 3). The mean time for complete epithelialization was significantly shorter in Group A (84.02 ± 1.76 days, 95% CI: 80.57, 87.48) compared to Group B (92.79 ± 1.50 days, 95% CI: 89.84, 95.73) with a log-rank p-value of 0.001. The difference was statistically significant at POD 45 (p < 0.0001) and POD 75 (p = 0.007). By POD 105, complete epithelialization was seen in 90.3% of patients in Group A vs. 95.2% in Group B, but this difference was insignificant (p = 0.473). The mean time for graft uptake was similar between Group A (76.94 ± 1.78 days, 95% CI: 73.44-80.43) and Group B (76.19 ± 1.78 days, 95% CI: 72.71-79.67) with a log-rank p-value of 0.794, and both groups achieving 100% graft uptake by POD 75.

No adverse events necessitating early pack removal were observed during any of the three 10-day packing periods, indicating that repeated medicated mastoid cavity packing is safe and well-tolerated in the postoperative setting.

## Discussion

The present study compared the efficacy of medicated mastoid cavity packing versus no packing during follow-up in patients undergoing CWD mastoidectomy, evaluating parameters such as discharge, granulation tissue formation, mucosal fold formation, epithelialization, and graft uptake. Our findings provide valuable insights into optimizing postoperative care strategies for patients with COM.

We noted early epithelialization in the medicated packing group compared to the no-pack group, evident at POD 45 (48.4% vs. 15.9%, p < 0.0001) and POD 75 (72.6% vs. 49.2%, p = 0.007). These findings suggest that medicated packing facilitates a conducive healing environment, likely by reducing microbial colonization and controlling excessive inflammatory responses, supported by previous research documenting enhanced healing with antimicrobial-impregnated packing materials [9]. Notably, the mean time to complete epithelialization was significantly shorter in the packed group (84.02 days) compared to the non-packed group (92.79 days), aligning well with the generally accepted re-epithelialization window of 2-3 months for CWD mastoidectomy cavities [13]. Since delayed healing beyond this period is associated with persistent ear discharge, our results highlight the potential benefit of medicated packing in reducing prolonged morbidity.

Similarly, granulation tissue formation was consistently higher in the no-pack group during the first six to eight postoperative weeks, though this difference did not reach statistical significance. By around three months, this complication had largely resolved in both groups. This trend suggests that medicated packing may support more efficient cavity remodeling by minimizing inflammation-driven granulation. Comparable conclusions were reported by Liu et al. (2023), who demonstrated reduced granulation formation using bacteriostatic packing in an animal model [9].

Regarding graft uptake, our study found no statistically significant differences between groups, with both achieving nearly complete graft uptake by POD 75. This observation indicates that while medicated mastoid cavity packing accelerates epithelialization, it does not significantly interfere with graft uptake or alter graft success rates. Javed et al. (2015) compared postoperative external ear canal packing with no packing in 32 adults undergoing primary posterior bony canal wall-preserving middle ear surgery and similarly reported comparable graft outcomes [10]. More recently, Turhal et al. (2024) evaluated packing with platelet-rich fibrin in endoscopic tympanoplasty and found no significant impact on graft success [12], reinforcing the notion that the type of packing may not critically influence graft uptake when the surgical technique is standardized. Nonetheless, differences in anatomical locations, surgical approaches, and packing materials emphasize the need for further comparative studies.

The incidences of mucosal fold formation were lower in the packed group during the early postoperative phase compared to the non-packed group, although the difference was not statistically significant over the three-month follow-up. This suggests that medicated mastoid packing does not contribute to excessive fibrosis or scarring. Previous studies have also demonstrated that adding anti-inflammatory agents, such as steroids or hyaluronic acid, to packing materials can reduce inflammation, adhesion, and new bone formation [14]. These findings support the potential role of medicated packing in modulating the postoperative inflammatory response and minimizing fibrotic changes. Importantly, no cases of meatal narrowing were observed in either group; however, extended follow-up is essential to assess potential late complications such as meatal stenosis.

An important clinical observation in our study was that postoperative discharge remained low and comparable between groups, with no statistically significant difference. Although the potential for packing materials to act as a medium for bacterial growth has been raised in previous studies [15], our findings support the notion that medicated mastoid packing does not increase the risk of discharge and can be safely used without contributing to postoperative infection. Furthermore, mastoidectomy with obliteration of the cavity is effective in preventing postoperative issues such as persistent discharge [16]. In our study, medicated mastoid packing may have provided a similar benefit through temporary cavity obliteration, helping stabilize the wound environment, reduce moisture retention, and limit bacterial colonization during the critical early healing period. This may partially explain the lower frequency of discharge observed in the packed group (3.2%) compared to 4.8% in the non-packed group.

Based on the study findings, medicated mastoid cavity packing offers meaningful clinical benefits and may serve as a valuable adjunct in the postoperative management of patients undergoing CWD mastoidectomy. Promoting earlier epithelialization can shorten the duration of otorrhea, reduce the need for topical and systemic antibiotics, and limit the frequency of follow-up visits. These benefits are significant in developing countries, where suboptimal hygiene and rising antibiotic resistance often compromise postoperative recovery. Such benefits enhance individual patient outcomes and alleviate the broader economic burden on healthcare systems, particularly in settings where access to ongoing care and resources is limited.

Medicated mastoid cavity packing offers meaningful clinical benefits, particularly by accelerating epithelialization and reducing postoperative discharge and formation of granulation, thereby serving as a useful adjunct in the postoperative management of patients undergoing CWD mastoidectomy. Importantly, repeated medicated mastoid cavity packing was found to be safe and well-tolerated, with no adverse events requiring early removal during the three packing cycles, further supporting its feasibility in routine postoperative care. However, this study has some limitations. First, although the sample size was adequate for detecting differences in epithelialization rates, it may not have been powered to detect subtler differences in other healing parameters. Second, the follow-up period of approximately three and a half months was relatively short, limiting the assessment of long-term outcomes and potential late complications. Third, while compliance was ensured through exclusion criteria, broader socioeconomic factors that may influence healing and access to care in the Indian context were not directly controlled. Future multicenter randomized controlled trials with larger cohorts, extended follow-up durations, and adjustments for socioeconomic variables are recommended to strengthen the evidence base. Additionally, studies should explore the long-term effects of medicated packing on hearing outcomes, disease recurrence, and patient-reported satisfaction.

## Conclusions

This study demonstrates that medicated mastoid cavity packing can significantly accelerate epithelialization following CWD mastoidectomy without adversely affecting graft uptake or increasing postoperative complications. By 6 weeks after surgery, about half of the patients who received packing had complete healing of the mastoid cavity, and nearly 9 out of 10 had successful graft uptake. Although differences in granulation tissue formation, mucosal fold presence, and discharge were not statistically significant, trends favored the packed group, suggesting potential benefits in early cavity remodeling and infection control. Medicated packing did not increase the risk of fibrosis, meatal narrowing, or delayed healing. These findings support the safe use of medicated mastoid packing as an effective adjunct in postoperative care and suggest broader health system benefits, such as reduced follow-up burden, improved patient adherence, and potential cost savings, particularly in resource-limited settings.
